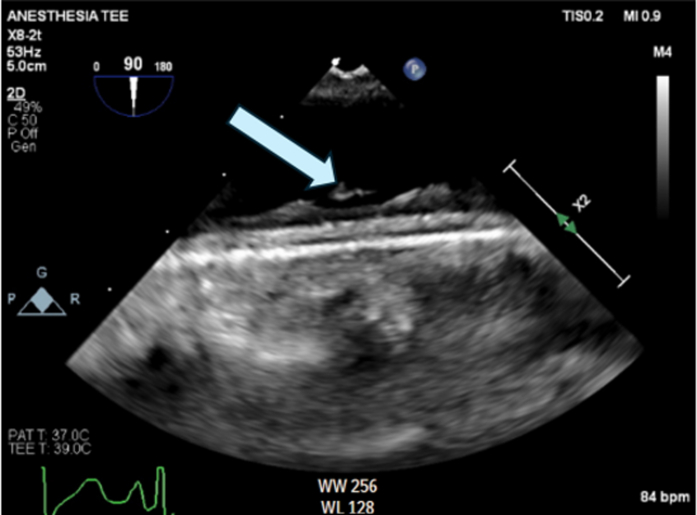# Anaortic Coronary Artery Bypass Grafting After Cardiovascular Collapse From Severe Syphilitic Aortitis With Coronary Obstruction

**DOI:** 10.1016/j.atssr.2025.01.005

**Published:** 2025-02-05

**Authors:** Nataly Montano Vargas, Danielle M. Mullis, Matthew Wingo, Alyssa C. Garrison, T. Robert Feng, John W. MacArthur

**Affiliations:** 1 Department of Cardiothoracic Surgery, Stanford University, Stanford, California; 2 Department of Anesthesiology, Stanford University, Stanford, California

## Abstract

This case report describes the rare case of cardiovascular collapse and coronary ostial stenosis secondary to syphilitic aortitis in a previously healthy 47-year-old woman. To avoid manipulation of a vasculitic aorta, anaortic coronary artery bypass grafting was performed. Syphilitic aortitis with coronary occlusive disease is rare since the advent of antibiotics, but this case report highlights the importance of including syphilitic aortitis on the differential diagnosis for coronary artery lesions.

Syphilitic aortitis is a rare complication of untreated tertiary syphilis, which is rare after the advent of antibiotics, although such cases have been noted since the late 1800s.^1^ This diagnosis tends to affect the aorta and its vascular tissue, most commonly resulting in aortic aneurysms, coronary stenosis, and aortic regurgitation.^2^ However, surgical management is not well established for patients with this pathology. In this case report, we describe the importance of such a diagnosis and the use of anaortic coronary artery bypass grafting (CABG) to successfully treat this patient.

A previously healthy 47-year-old woman from Mexico presented to the emergency department with sudden onset of chest pain and upper back pain with associated nausea and vomiting for 1 day. Upon arrival to the hospital, her troponin was elevated, and electrocardiography showed diffuse T-wave inversions with anterior ST-elevations suggestive of ischemia (Figure 1). Chest computed tomography ruled out aortic dissection. She was immediately brought to the catheterization laboratory, where she was found to have 99% occlusion of the right coronary artery (Supplemental Video 1), which was unable to be engaged for percutaneous coronary intervention. Left-sided lesions were not initially identified in the cardiac catheterization laboratory.

**Figure 1:**
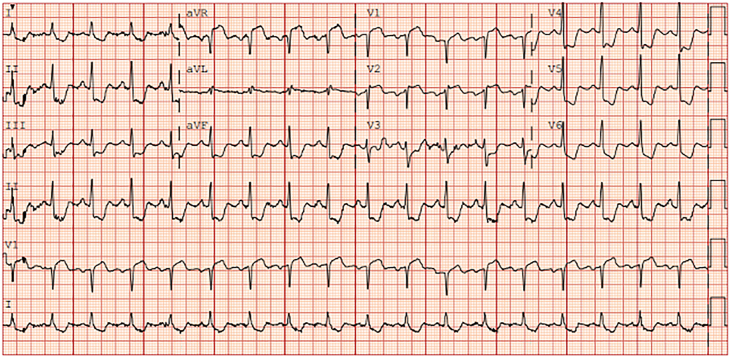
A 12-lead electrocardiogram taken upon emergency department arrival shows ST-elevation and diffuse T-wave inversions suggestive of ischemia.

Complete heart block subsequently developed, and she went into bradycardic arrest requiring cardiopulmonary resuscitation and chest compressions. Venoarterial extracorporeal membrane oxygenation (VA-ECMO) was emergently initiated, and an Impella CP (Abiomed*)* and transvenous pacemaker were inserted. She returned to sinus rhythm and was taken to the intensive care unit.

The patient was transferred to the cardiac intensive care unit for further monitoring and surgical planning. Transthoracic echocardiography revealed severely reduced systolic function, with an estimated left ventricular ejection fraction of 0.18 (Supplemental Video 2) and moderate aortic regurgitation. Without revascularization, the team felt there was no option to separate the patient from mechanical circulatory support. Transplantation was considered, but she was not a candidate because she was found to have advanced syphilis. Therefore, surgical revascularization was her only treatment option.

Cardiac-gated computed tomography coronary angiography with intravenous contrast demonstrated circumferential wall thickening of the ascending aorta and aortic root (Figure 2A) with nearly complete occlusion of the ostia of the right coronary artery (Figure 2B) and left main coronary artery (Figure 2C; Supplemental Video 3). Anaortic CABG was planned.

**Figure 2:**
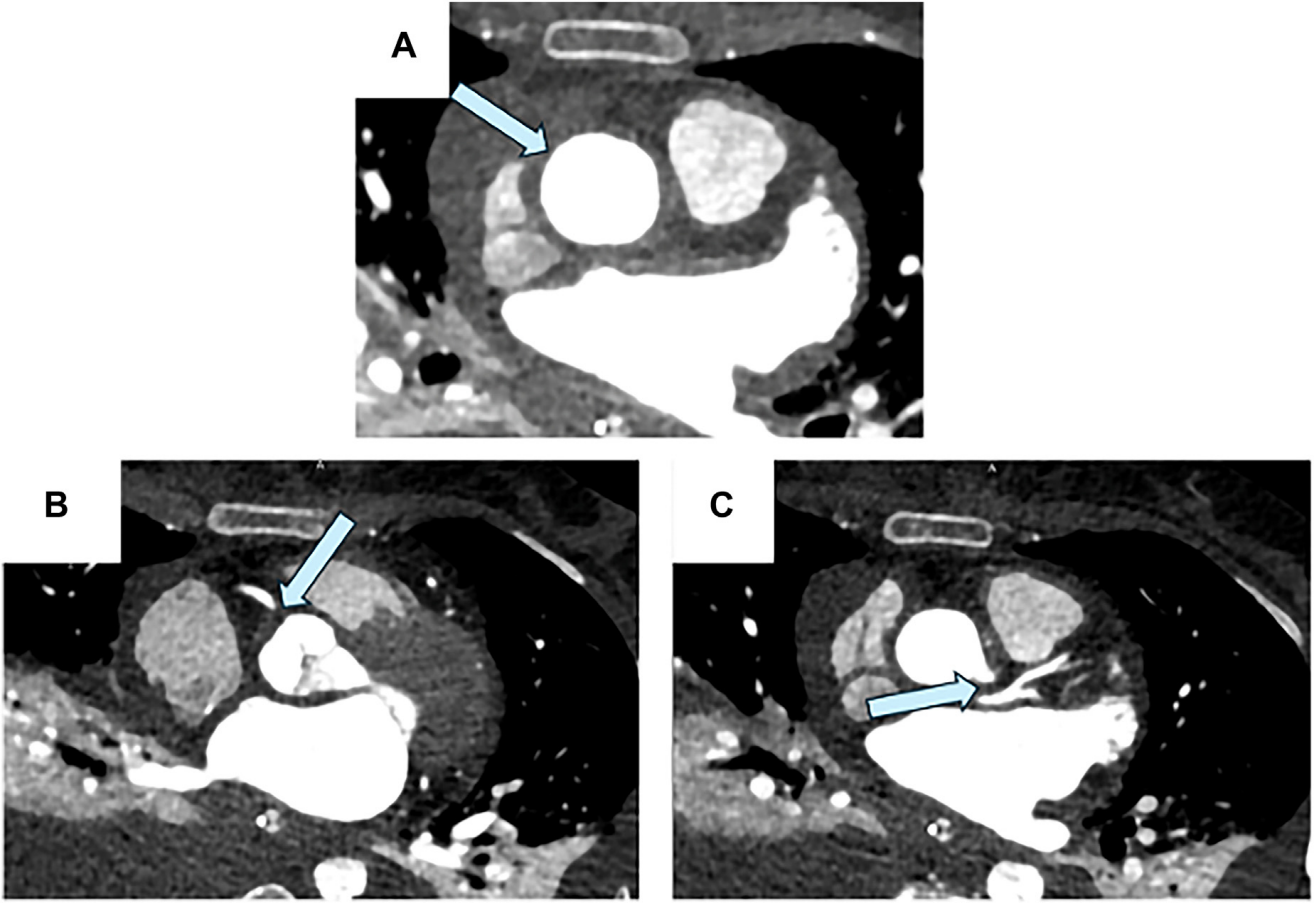
Preoperative retrogated computed tomography reveals coronary ostial stenosis secondary to syphilitic aortitis. Arrows show (A) circumferential wall thickening of the ascending aorta, (B) nearly complete occlusion of the right coronary artery, and (C) nearly complete occlusion of the left coronary artery.

The patient was brought to the operating room and a midline sternotomy was performed. The heart was rotated and stabilized with the Octopus device (Medtronic). An end-to-side anastomosis was performed between the left anterior mammary artery and the left anterior descending artery with 7-0 Prolene (Ethicon) suture. Another end-to-side anastomosis was performed between the right anterior mammary artery and the distal right coronary artery with 7-0 Prolene suture. The Impella CP was removed, the chest was packed and left open, and the patient was taken to the intensive care unit in stable condition.

On postoperative day 2, the patient’s chest was closed, and she was weaned off VA-ECMO on postoperative day 9. Intraoperative transesophageal echocardiography during the VA-ECMO decannulation revealed thickening and contracture of the aortic cusps (Figure 3; Supplemental Video 4) and atherosclerosis in the descending aorta with mobile plaque (Supplemental Figure) that was also observed during the CABG. She then completed a 2-week course of intravenous penicillin.

**Figure 3:**
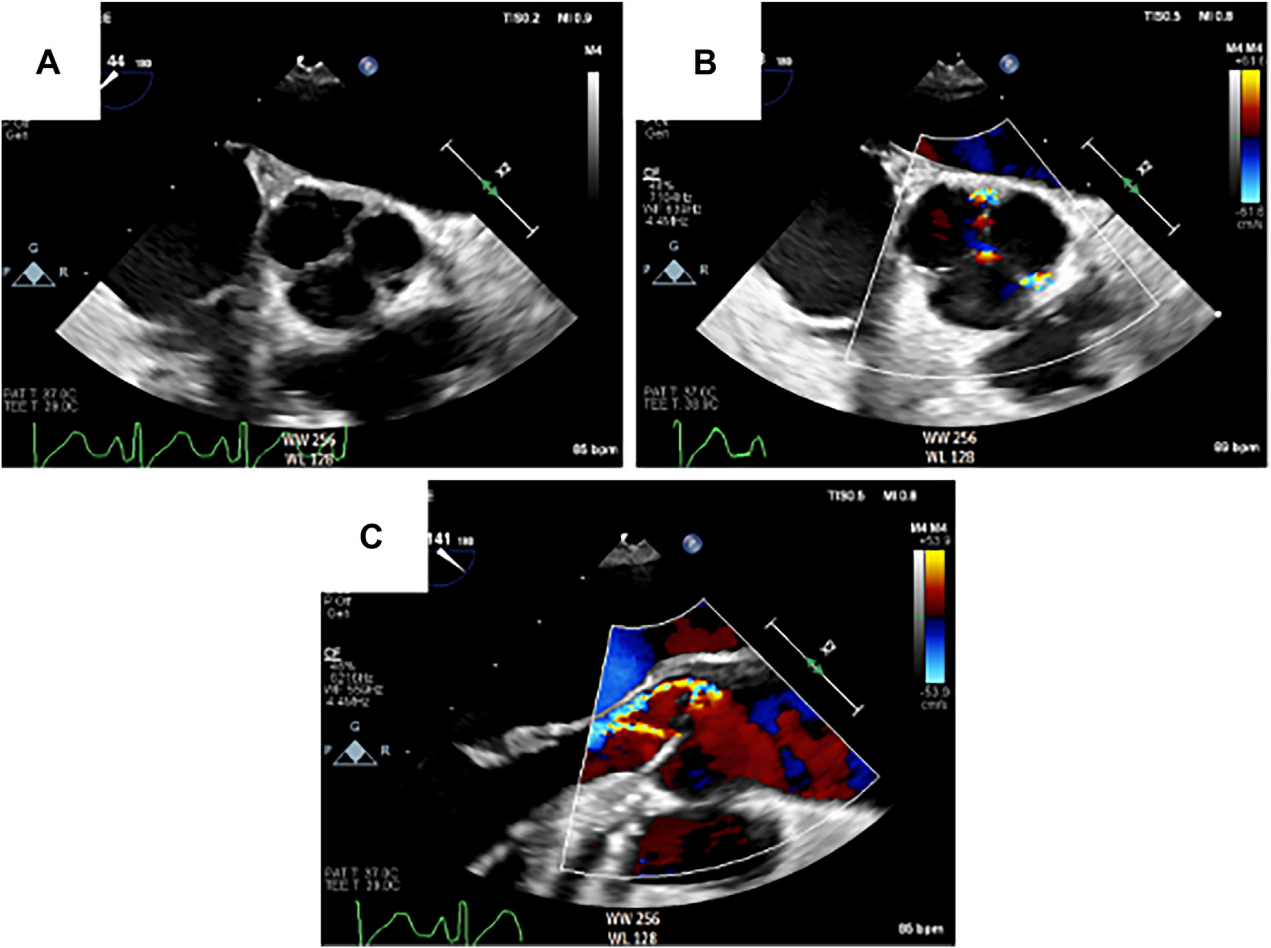
Intraoperative transesophageal echocardiography of the syphilitic aortic valve. (A) Midesophageal short-axis view of the aortic valve demonstrates thickening and contracture of aortic cusps. (B) Midesophageal short-axis view of the aortic valve with color Doppler shows central aortic regurgitation and aortic regurgitation at the left-non and left-right commissures. (C) Midesophageal long-axis view of the aortic valve with color Doppler.

Follow-up computed tomography angiography with intravenous contrast demonstrated 2 patent bypass grafts (Supplemental Video 5). As of 6 months after surgery, the patient’s ejection fraction had improved to ∼0.49 (Supplemental Video 6), and she had not been readmitted to the hospital and remained in good health.

## Comment

Severe disease later develop in ∼ 25% to 40% of patients with untreated syphilis.^3^ Symptoms may appear anywhere from 1 to 30 years after a primary infection.^3^ Local tissue destruction is present at this stage due to the immune system’s T cells fighting the infection. Possible lesions can include syphilitic aortitis, gumma formation, and neurologic syphilis. Syphilitic aortitis develops from long-term exposure to the spirochete *Treponema pallidum*, which over time damages the vasa vasorum causing vasculitis of the aortic wall.^4^ Syphilis can also more rarely affect the coronary arteries, causing coronary ostial stenosis secondary to syphilitic aortitis.^5^ Clinical significance of tertiary syphilitic aortitis rests in its potential to cause severe cardiovascular complications if not diagnosed and treated effectively.

Our case report underscores the importance of including tertiary syphilis in a patient's differential diagnosis as a cause for coronary artery lesions. Previous reports have demonstrated successful patient outcomes with similar surgical treatments.^6^, ^7^, ^8^ This is a rare occurrence of a patient who presented with cardiovascular collapse due to bilateral coronary artery ostial occlusions leading to mechanical circulatory support and was treated with high-risk surgical revascularization. Furthermore, we elected to use an anaortic technique for surgical revascularization to avoid manipulation of a vasculitic aorta. Although syphilitic aortitis with coronary occlusive disease may be rare, it is important to apply innovative surgical strategies to optimize patient outcomes.

## Figures and Tables

**Supplementary Figure 1:**