# Supplementary material for: Anaortic Coronary Artery Bypass Grafting After Cardiovascular Collapse From Severe Syphilitic Aortitis With Coronary Obstruction
Source: Ann Thorac Surg Short Rep. 2025 Feb 5;3(3):576–9. doi: 10.1016/j.atssr.2025.01.005 (PMC12559270; doi:10.1016/j.atssr.2025.01.005)
Supplement: Supplementary Material [file mmc1.docx]

**Supplement Figure 1: Intraoperative Transesophageal Echocardiogram Demonstrates Mobile Plaque in Descending Aorta.**
